# Cocaine Modulates the Neuronal Endosomal System and Extracellular Vesicles in a Sex-Dependent Manner

**DOI:** 10.1007/s11064-022-03612-1

**Published:** 2022-04-30

**Authors:** Bryana R. Barreto, Pasquale D’Acunzo, Jonathan M. Ungania, Sasmita Das, Audrey Hashim, Chris N. Goulbourne, Stefanie Canals-Baker, Mitsuo Saito, Mariko Saito, Henry Sershen, Efrat Levy

**Affiliations:** 1 Center for Dementia Research, Nathan S. Kline Institute for Psychiatric Research, Orangeburg, NY 10962 USA; 2 Department of Psychiatry, New York University Grossman School of Medicine, New York, NY 10016 USA; 3 Division of Neurochemistry, Nathan S. Kline Institute for Psychiatric Research, Orangeburg, NY 10962 USA; 4 Department of Biochemistry & Molecular Pharmacology, New York University Grossman School of Medicine, New York, NY 10016 USA; 5 NYU Neuroscience Institute, New York University Grossman School of Medicine, New York, NY 10016 USA

**Keywords:** Endosome, Extracellular vesicle, Exosome, Chronic drug exposure, α-synuclein, Cocaine

## Abstract

In multiple neurodevelopmental and neurodegenerative disorders, endosomal changes correlate with changes in exosomes. We examined this linkage in the brain of mice that received cocaine injections for two weeks starting at 2.5 months of age. Cocaine caused a decrease in the number of both neuronal early and late endosomes and exosomes in the brains of male but not female mice. The response to cocaine in ovariectomized females mirrored male, demonstrating that these sex-differences in response to cocaine are driven by hormonal differences. Moreover, cocaine increased the amount of α-synuclein per exosome in the brain of females but did not affect exosomal α-synuclein content in the brain of males, a sex-difference eliminated by ovariectomy. Enhanced packaging of α-synuclein into female brain exosomes with the potential for propagation of pathology throughout the brain suggests a mechanism for the different response of females to chronic cocaine exposure as compared to males.

## Introduction

Cocaine is presently one of the most abused stimulant drugs in the United States. It binds to and inhibits the sodium-dependent dopamine (DA) transporter (Slc6a3, also known as DAT), increasing the levels of DA in the extracellular space. Beyond the known effects of cocaine on DAT, long-term repeated cocaine use has actions independent of DAT, including an effect on α-synuclein-related pathways. Repeated cocaine use elevates α-synuclein levels in blood and brain tissue in humans and increased levels of α-synuclein correlate with increased cocaine cravings [1, 2]. Multiple studies using α-synuclein overexpressing or KO mice argue that α-synuclein is involved in the regulation of DA neurotransmission [3–6]. Postmortem human studies show higher levels of α-synuclein in ventral tegmental area and substantia nigra dopaminergic neurons of cocaine addicts [3]. Increased levels of α-synuclein result in a direct insult to the dopaminergic system [3]. Furthermore, cocaine has a direct effect on the D2 and D1 receptors and a direct action on the remodeling of lipid rafts [7–9] that serve as a platform for regulating receptors and other proteins including DAT [10]. Lipid rafts are an important pathway for clathrin-independent endocytosis [11], suggesting an effect of cocaine on the endosomal system. In fact, cocaine induces the formation of large vacuoles in cells in vitro, likely via fusion of late endosomes, and these enlarged vacuoles can trigger dysregulation of the endocytic flux, leading to damage to the cell [12]. Upon repeated administration, cocaine causes alterations in the endocytic [6], autophagic [13], and lysosomal compartments [14].

Intraluminal vesicles are formed by the invagination of the late endosome/multivesicular body (MVB) membrane around cytoplasmic materials. MVB content can either be delivered to the lysosome for degradation or secreted by the cell, with the intraluminal vesicles now known as exosomes. Exosomes have diverse biological properties, markers, and functions, and are of research interest as long-lived vesicles often found distant from the exosome generating cell that can carry important information regarding the source cell and the endosomal-lysosomal system within that cell [15–18]. Our prior studies have shown that modifications of the endocytic system are directly linked to a change in the biogenesis of extracellular vesicles (EVs) in the brain [15, 19–22]. EVs are nanoscale secreted vesicles that encapsulate lipids, proteins, and nucleic acids and are involved in cell-to-cell communication, waste removal, and transfer of bioactive molecules between cells. Classically, two main EV subpopulations of different origin have been defined, called microvesicles and exosomes. Each of these EV populations are secreted by diverse cell types, are found in all body fluids, and have specific biological properties, markers, and functions [15–18]. Given the effect of cocaine on the endosomal-lysosomal system, we hypothesized that exosomes within the cocaine-treated brain likely reflect alterations within the neuronal endosomal-lysosomal system, and that altered regulation of the exosomal system may contribute to the neurobiological mechanisms of chronic cocaine exposure.

Thus, we investigated EVs in the brain extracellular space of mice in response to chronic, non-contingent cocaine treatment. Male and female mice were included in the study because we have previously identified sex differences in drug-induced responses [23]. By using a recently described approach to isolate and separate EVs [24], we show that cocaine has a sex-dependent impact on exosomes levels and cargo. We provide evidence that cocaine perturbs EV secretion and the endocytic pathway in vivo in neurons of male, but not female, mice, suggesting an underlining role of gonadal hormones in chronic cocaine exposure mechanisms. These studies may lead to find sex-specific therapies for substance use disorder and/or biomarkers for chronic cocaine exposure.

## Materials and Methods

### Experimental Design and Statistical Analyses

Male and female C57BL/6 J mice (indicated as wild-type mice in the text, RRID:IMSR_JAX:000,664) were purchased from the Jackson Laboratory (Bay Harbor, ME, US). Starting at 2.5 months of age, a cohort of C57BL/6 J male and female mice were given non-contingent intraperitoneal injection of 10 mg/kg cocaine-HCl (Sigma-Aldrich, St. Louis, MO, US) prepared in sterile 0.9% sodium chloride solution (saline; Hospira, Lake Forest, IL, US) or saline as control, once daily for 12 days [25]. An additional cohort of female 2-month-old C57BL/6 J mice were subjected to ovariectomy or sham surgery. Under isoflurane anesthesia and sterile field, a dorsal transverse incision was made to allow bilateral access to both ovaries. The abdominal wall was cut over each white fatty tissue, ovaries retracted and cut, and the muscle layer and skin sutured. The mice rested for 14 days post-operation and then were administered non-contingent intraperitoneal injection of cocaine or saline, once daily for 12 days. Mice were sacrificed 30 min after the final cocaine/saline injection.

For biochemical analyses of mouse brains, mice were sacrificed by cervical dislocation and the two hemibrains without the cerebellum and the olfactory bulbs were kept at − 80 °C until further processing. For immunohistochemical procedures, mice were anesthetized with isoflurane (Henry Schein Animal Health, Melville, NY, US) and transcardially perfusion-fixed with 4% paraformaldehyde (PFA, Electron Microscopy Sciences, Hatfield, PA, US) in phosphate buffered saline (PBS, Corning Incorporated, Corning, NY, US). Brains were removed and post-fixed overnight in 4% PFA in PBS at 4 ºC, transferred to 20% glycerol/2% DMSO/0.1 M phosphate buffer (all reagents from Sigma-Aldrich) on the next day and kept at 4 ºC until further processing. All experiments were performed following the ‘Animal Research: Reporting In Vivo Experiments’ (ARRIVE) guidelines. Statistical analysis was carried out using GraphPad Prism (version 6.01, GraphPad Holdings, San Diego, CA, US). Data are shown as mean ± standard error of the mean (SEM). The variable *n* is defined as the number of mice analyzed per experimental condition. It corresponds to the number of mice within each group for behavior and immunohistochemistry data. EVs were isolated from one mouse hemibrain for each experimental group per each isolation, and four hemibrains were manipulated together: male/female either treated with saline or cocaine, or sham/ovariectomized females treated with saline or cocaine. For immunohistochemistry, the average of 30 random neurons in the frontoparietal cortex was calculated for each mouse, and we included 4 different mice per group (n = 4), for a total of ~ 120 neurons per experimental group. ImageJ was used to quantify the number, area, and diameter of endosomes using the “Analyze Particles” plug-in in a fully unbiased, automatized way. Significance was calculated through two-way ANOVA with Bonferroni’s multiple comparisons test, considering as significant changes with a *P* < 0.05 (95% confidence interval). All experiments were performed at least three times independently, and mice were randomly allocated to saline or cocaine groups, as well as to either sham surgery or ovariectomy. To minimize statistical confounders, cage location and the order of treatments were randomly allocated. No data points were excluded by the analysis and no criteria were set to exclude animals or data points.

### Behavioral Assay

Locomotor activity was measured with Opto-Varimex activity monitors (Columbus Instruments, Columbus, OH, US), and calculated based on total ambulatory counts (TAC) of consecutive beams broken during ambulation. Single beams broken repeatedly were not counted. Data are expressed as TAC over 60 min after the injection of cocaine on days 1, 8 and 11.

### Immunohistochemistry

Brains were cut into 40 µm-thick coronal sections with a vibratome. Free-floating sections from all mouse groups were concurrently processed for immunohistochemical examination [26]. Control sections were processed with the omission of either the primary or secondary antibodies to exclude non-specific reactions. Labeling conditions and exposure times were identical throughout.

Fluorescent labeling of early and late endosomes was performed using antibodies to Rab5a (1:100, clone EPR21801, ab218624, Abcam, Cambridge, UK, RRID:N/A) and Rab7a (1:100, clone 5G8.1, MABC119, EMD Millipore, Billerica, MA, US, RRID:N/A), respectively. Double immunolabeling with antibodies to the neuronal nuclei antigen (NeuN, also known as Rbfox3) was performed to identify neurons. We used an anti-NeuN mouse monoclonal antibody (1:100, clone A60, MAB377, EMD Millipore, RRID:AB_2298772), for the double staining with Rab5a and an anti-NeuN rabbit polyclonal antibody (1:100, ABN78, EMD Millipore, RRID:AB_10807945) for the double staining with Rab7a. Following incubation with fluoresceinated secondary antibodies (A21202 donkey, AlexaFluor488-conjugated, anti-mouse secondary antibody, RRID:AB_141607; A21206 donkey, AlexaFluor488-conjugated, anti-rabbit secondary antibody, RRID:AB_2535792; A11031 goat, AlexaFluor568-conjugated, anti-mouse secondary antibody, RRID:AB_144696; A11036 goat, AlexaFluor568-conjugated, anti-rabbit secondary antibody, RRID:AB_10563566; all antibodies from ThermoFisher Scientific, Waltham, MA, US, and used at a 1:500 dilution). Slides were covered with coverslips using an aqueous mounting medium designed to preserve fluorescence (Fluoroshield, Sigma-Aldrich). Immunofluorescence was observed and captured using an LSM 510 Meta confocal microscope (Zeiss, Thornwood, NY, US). For quantification, we calculated the number of endosomes and endosome diameter as the average of at least 30 random neurons in the frontoparietal cortex per mouse, and we included 4 different mice per group (n = 4), for a total of ~ 120 neurons per experimental group. We chose to quantify cortical pyramidal neurons instead of midbrain DA neurons because cortical pyramidal neurons are the more abundant neuronal population found in a total murine hemibrain and, as such, endosomal dynamics in these cells are more representative of our EV data (EVs are isolated from a whole right hemibrain). Data were measured by a treatment- and surgery-blinded observer. Quantification of Rab5a and Rab7a signal was performed using ImageJ (NIH, Bethesda, MD, US) [27]. Briefly, ImageJ was first calibrated for each picture by adjusting to the scale on each image. ImageJ “Watershed” plug-in was used to separate overlapping particles in binary images. ImageJ quantified the number, area, and diameter of particles using the “Analyze Particles” plug-in in a fully unbiased, automatized way. To ensure the software would neglect aberrant background, the exclusion parameter was set to diameter < 100 nm. Two-way ANOVA followed by post-hoc multiple comparisons Bonferroni’s test was used to assess the differences between groups (variables considered: sex or surgery and treatment); level of statistical significance was set at *P* < 0.05.

### Brain Homogenates

Frozen left hemi-brains were weighed and stored at − 80 °C until homogenization. Protease inhibitors [5 μg/mL leupeptin, 5 μg/mL antipain dihydrochloride, 5 μg/mL pepstatin A, 1 mM phenylmethanesulfonyl fluoride (PMSF), 1 μM E64; all reagents from Sigma-Aldrich] were added to a tissue homogenization buffer (THB: 0.25 M sucrose, 20 mM Tris–HCl pH 7.4, 1 mM EDTA, 1 mM EGTA; all reagents from Sigma-Aldrich) immediately before homogenization. Brains were homogenized in an ice-cold glass homogenizer with a Teflon pestle (Wheaton, DWK Life Sciences, Millville, NJ, US) in 10% *v/w* THB with 20 complete up-and-down spinning strokes. Aliquots of homogenate were stored at − 80 °C until use.

### Isolation of EVs From Brain Parenchyma

Murine EVs were isolated from the right hemibrains as previously described [28, 29]. Briefly, hemibrains were minced and incubated with 20 U/mL papain (Worthington, Lakewood, NJ, US) in Hibernate A (BrainBits, Springfield, IL, US) for 15 min at 37 °C. The enzymatic digestion was stopped by the addition of ice-cold protease inhibitors in Hibernate A. The solution was gently disassociated by pipetting and centrifuged at 300* g* for 10 min at 4 °C. The supernatant was subsequently filtered twice, first through a 40 μm cell strainer (Fisher Scientific, Pittsburgh, PA, US) and then through a 0.2 μm surfactant-free cellulose acetate (SFCA) syringe filter (Corning Incorporated). The cleared mixture was centrifuged at 2,000* g* for 10 min at 4 °C and the supernatant centrifuged at 10,000* g* for 30 min at 4 °C. The supernatant was then ultra-centrifuged at 100,000* g* (*k*-factor: 207.5, 45Ti rotor type, Beckman Coulter, Brea, CA, US) for 70 min at 4 °C. The pellet was washed once in PBS pH 7.4 (Corning Incorporated), re-centrifuged at 100,000* g* for 70 min at 4 °C and resuspended in a 40% *v/v* OptiPrep (Sigma-Aldrich) solution, containing 10 mM Tris–HCl pH 7.4, 0.25 M sucrose and 40% iodixanol (all reagents from Sigma-Aldrich) for fractionation on an OptiPrep density step-gradient [24]. The gradient was set up by carefully layering on the top of the 40% OptiPrep-equilibrated EVs a decreasing scale of OptiPrep solutions (20%, 15%, 13%, 11%, 9%, 7%, 5%). The column was centrifuged overnight at 4 °C in a swinging bucket rotor at 200,000* g* (*k*-factor: 137.3, SW40Ti rotor type, Beckman Coulter). Afterwards, 1.5 mL fractions, corresponding to the different interphases, were collected, washed in PBS, and centrifuged at 100,000* g* for 70 min at 4 °C in a fixed-angle rotor (*k*-factor: 61.2, MLA80 rotor type, Beckman Coulter). Pellets were resuspended in PBS. The strategy employed in this study to isolate brain EVs is designed to enrich for canonical small EVs, including microvesicles, exosomes, and mitovesicles. It does not isolate all types of EV-like particles that may be present in the brain. Apoptotic bodies and very large oncosomes (> 1 µm) are filtered and pelleted out by the first steps of our brain EV isolation procedure and therefore are not included in the crude brain EV pellet. On the other hand, very small (< 50 nm) EV-like particles, including exomeres, pellet only by very long (16 h) centrifugation steps at 167,000* g* [30], while we obtain the precolumn brain EV pellet using only a 100,000* g* centrifugation step for 70 min. Accordingly, exomeres are not pelleted using our conditions and are discarded together with the supernatant of the first 100,000* g* centrifugation. BCA protein assay, transmission electron microscopy and nanotrack analysis (NTA) of brain EVs were performed using manufacturer’s instructions, as described in detail elsewhere [24]. For Western blot analyses, aliquots were lysed adding 1:1 equal volume of 2X RIPA buffer (2% Triton X-100, 2% sodium deoxycholate, 0.2% SDS, 300 mM sodium chloride, 100 mM Tris–HCl pH 7.4, 2 mM EDTA, all reagents from Sigma-Aldrich) with protease inhibitors. The characterization of the EVs shown in this paper comply in full with the minimal information for studies of EVs 2018 (MISEV2018) guidelines [31].

### Western Blot Analysis

EV fractions and brain homogenates were supplemented with a 6X Laemmli sample buffer (375 mM Tris–HCl pH 7.4, 9% SDS, 50% glycerol, 9% β-mercaptoethanol, 0.03% bromophenol blue, all reagents from Sigma-Aldrich) and boiled for 5 min at 95 °C. Equal volumes for EV preparations or equal amount of proteins for brain homogenates were loaded on 4–20% gradient precast Tris–HCl polyacrylamide gels (Bio‐Rad, Hercules, CA, US) and ran for 2 h at 120 V. Samples were then transferred onto a PVDF membrane (Immobilon, EMD Millipore) overnight at 100 mA. The membranes were blocked in 5% *w/v* BSA (Sigma-Aldrich) for 1 h. Primary antibodies used in this study were: anti-ALG-2-interacting protein X (Alix, also known as Pdcd6ip, 1:1,000, #92880S, Cell Signaling Technology, Danvers, MA, US, RRID:AB_2800192), anti-heat shock cognate 71 kDa protein 8 (Hsc70, also known as Hspa8, 1:1,000, #sc-7298, Santa Cruz Biotechnology, Santa Cruz, CA, US, RRID:AB_627761), anti-Cd63 (1:1,000, #ab217345, Abcam, RRID:AB_2754982), anti-Annexin A2 (1:10,000, #ab178677, Abcam, RRID N/A), anti-α-synuclein (1:1,000, #610,787, BD Biosciences, San Jose, CA, US, RRID:AB_398108), anti-Rab27a (1:1,000, #ab55667, Abcam, RRID:AB_945112), anti-Rab35 (1:1,000, #9690, Cell Signaling Technology, RRID:AB_11178805), anti-130 kDa cis-Golgi matrix protein (Gm130, also known as Golga2, 1:300, #610,823, BD Biosciences, RRID:AB_398141), anti-β-actin (1:30,000, #3700, Cell Signaling Technology, RRID:AB_2242334), anti-cytochrome c oxidase subunit 4, isoform 1 (Cox-IV, also known as Cox4i1, 1:1,000, #ab33985, Abcam, RRID:AB_879754). The secondary antibodies used were HRP-conjugated from Jackson ImmunoResearch (West Grove, PA, US). Membranes were incubated with ECL (Pierce, Rockford, IL, US) or femto ECL (Pierce) for 5 min and protein bands were visualized with the iBright FL1500 imaging system (ThermoFisher Scientific). Protein bands were quantified using ImageJ.

## Results

### Behavioral Locomotion is Affected by Cocaine Administration in a Sex- and Hormone-Dependent Fashion

**Fig. 1:**
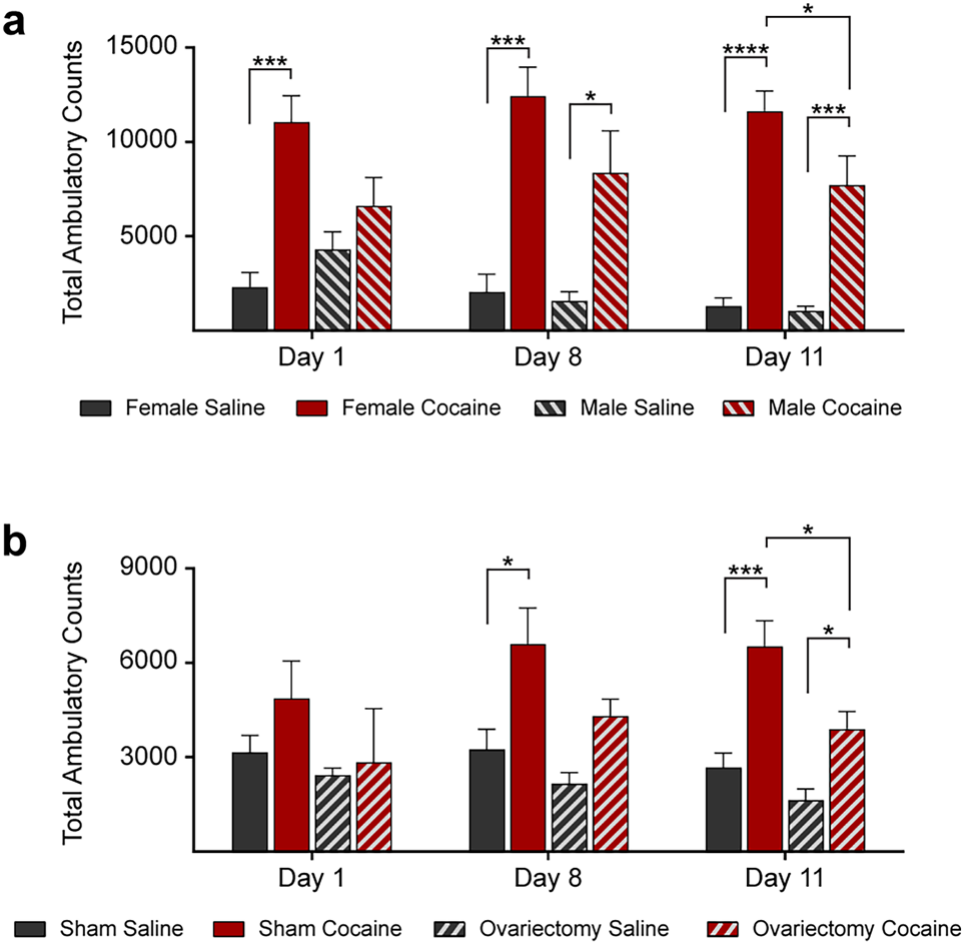
Cocaine-induced locomotor activity is sex- and hormone-dependent. **a** Saline or cocaine (10 mg/kg, i.p.) were injected 30 min after the mouse was placed in the activity monitor. Mice were treated once daily for 12 days and total ambulatory counts were measured for 60 min after each injection (days 1, 8, and 11 are shown). Females responded to cocaine injections with higher locomotor activity as compared with males (*n* = 6 mice per group). **b** Ovariectomy reduced the cocaine-induced locomotor activity of female mice (*n* = 6 mice per group). Statistical test: two-way ANOVA with Bonferroni’s multiple comparisons test. Data are represented as mean ± SEM. The differences between the groups were significant at * *P* < 0.05, *** *P* < 0.001 and **** *P* < 0.0001

As an animal model for substance use disorder, cocaine administration to mice has been widely used to study behavioral, molecular, and structural parameters associated with human processes related to chronic drug exposure. C57BL/6 J mice (2.5-month-old) were injected with non-contingent doses of either cocaine (10 mg/kg) or saline once daily for 12 days [25], and locomotor activity was measured and calculated based on TAC (total ambulatory counts) over 60 min post injection. The mice displayed enhanced locomotor activity after repeated cocaine administration (Fig. 1a). Although drug use is higher in men, women appear to be more prone to develop drug dependence, suffer more severe physical and psychological consequences of drug abuse, and have more difficulties in quitting [32]. We observed a greater locomotor activity response in female C57BL/6 mice than males (Fig. 1a), consistent with previous reports [23]. In order to investigate the hormonal effect in the response of females to cocaine as compared with males, females were ovariectomized prior to the chronic injection of cocaine or saline. Similar to males, ovariectomized females had less locomotor activity response to cocaine than sham treated female mice (Fig. 1b).

### Cocaine Impacts the Endosomal Pathway Differently in Males and Females

**Fig. 2:**
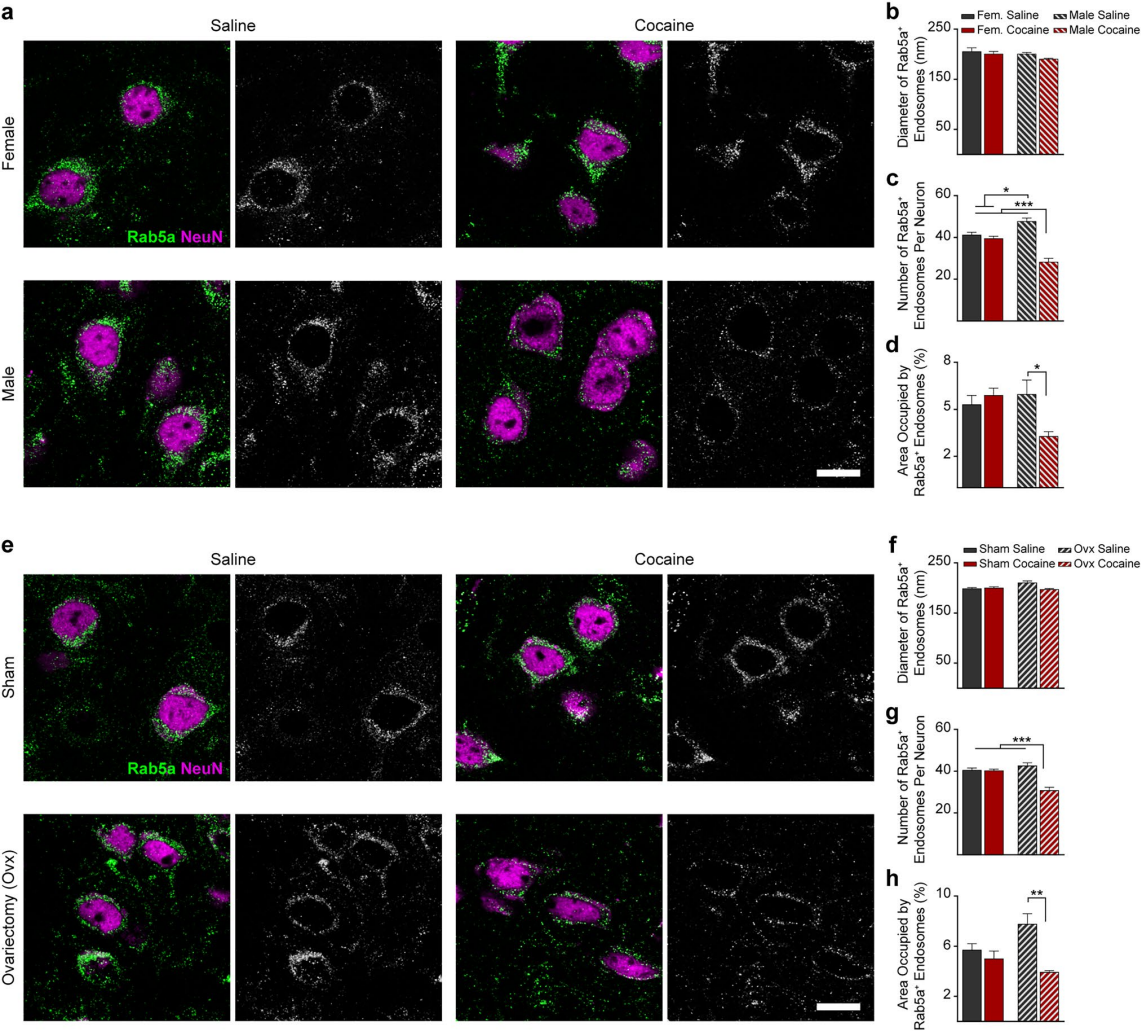
Cocaine affects Rab5-postitive early endosomes in a sex-dependent manner. **a**–**d** Neurons in the frontoparietal cortex of cocaine-treated male mice show lower number and area fraction per neuron of Rab5a-positive early endosomes as compared with saline-treated male mice. No effect was found in the brains of female mice (*n* = 4 mice per group; for each mouse, the average of at least 30 neurons was calculated). **e**–**h** Similar to males, in ovariectomized females, cocaine caused lower number and area fraction per neuron of early endosomes as compared with sham-treated females (*n* = 4 mice per group; for each mouse, the average of at least 30 neurons was calculated). **a** and **c** Double immunolabeling with antibodies to Rab5a (green) and NeuN (fuchsia) and grayscale Rab5a staining in cortical neurons are shown (scale bars, 10 µm). **b** and **f** Diameter, **c** and **g** Number, **d** and **h** Area per neuron of Rab5a-labeled endosomes. Statistical test: two-way ANOVA with Bonferroni’s multiple comparisons test. Data are represented as mean ± SEM. The differences between the groups were significant at * *P* < 0.05, ** P < 0.01, and *** *P* < 0.001

**Fig. 3:**
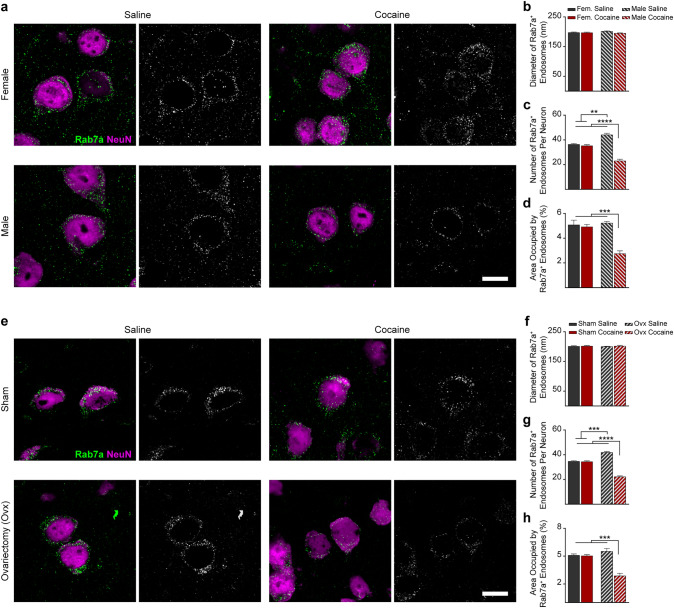
Cocaine affects Rab7-positive late endosomes in a sex-dependent manner. **a**–**d** Neurons in the frontoparietal cortex of cocaine-treated male mice show lower number and area fraction per neuron of Rab7a-positive late endosomes as compared with saline-treated male mice. No effect was found in the brains of female mice (*n* = 4 mice per group; for each mouse, the average of at least 30 neurons was calculated). **e**–**h** Similar to males, in ovariectomized females, cocaine caused lower number and area fraction per neuron of late endosomes as compared with saline treated ovariectomized females (*n* = 4 mice per group; for each mouse, the average of at least 30 neurons was calculated). **a** and **e** Double immunolabeling with antibodies to Rab7a (green) and NeuN (fuchsia) and grayscale Rab7a staining in cortical neurons are shown (scale bars, 10 µm). **b** and **f** Diameter, **c** and **g** Number, **d** and **h** Area per neuron of Rab7a-labeled endosomes. Statistical test: two-way ANOVA with Bonferroni’s multiple comparisons test. Data are represented as mean ± SEM. The differences between the groups were significant at ** P < 0.01, *** P < 0.001, and **** P < 0.0001

For morphological examination of early and late endosomes, murine frontoparietal cortex sections were labeled as previously described [26] either with an antibody against the small GTPase Rab5a, a regulator of endocytosis and a specific marker of early endosomes [33] (Fig. 2), or with an antibody to Rab7a, a regulator of vesicular trafficking in the late endocytic pathway and a late endosomal marker [34] (Fig. 3). Double staining with antibodies to the neuronal nuclei protein NeuN was used to identify neuronal endosomes. Rab5a- and Rab7a-immunolabelling in random frontoparietal cortical neurons were quantified to compare the endosomal diameter, number of endosomes per neuron, and average endosomal area per neuron of early and late endosomes among the mouse groups. While no effect of cocaine was found on Rab5a-positive early endosomes in cortical neurons of female mice, a significantly lower number and area fraction of early endosomes was found in cocaine-injected compared to saline-injected males, with no effect on endosomal size (Fig. 2a–d). Similarly, immunostaining with anti-Rab7a antibody revealed no effect of cocaine on late endosomes in cortical neurons of female mice but a significant lower number and area fraction of late endosomes in cocaine-injected compared to saline-injected males (Fig. 3a–d). Similar to males, in ovariectomized females, cocaine caused lower number and area fraction per neuron, but did not affect the apparent size of early (Fig. 2e–h) and late endosomes (Fig. 3e–h) as compared with sham treated females. Our data also show that the number of both early and late endosomes in brain neurons of male mice is higher than of female mice (Figs. 2, 3). Similar to males, in ovariectomized females the number of Rab7-positive neuronal endosomes is higher as compared to sham treated females (Fig. 3g).

### Cocaine Impacts the Number of Brain-Derived EVs Differently in Males and Females

**Fig. 4:**
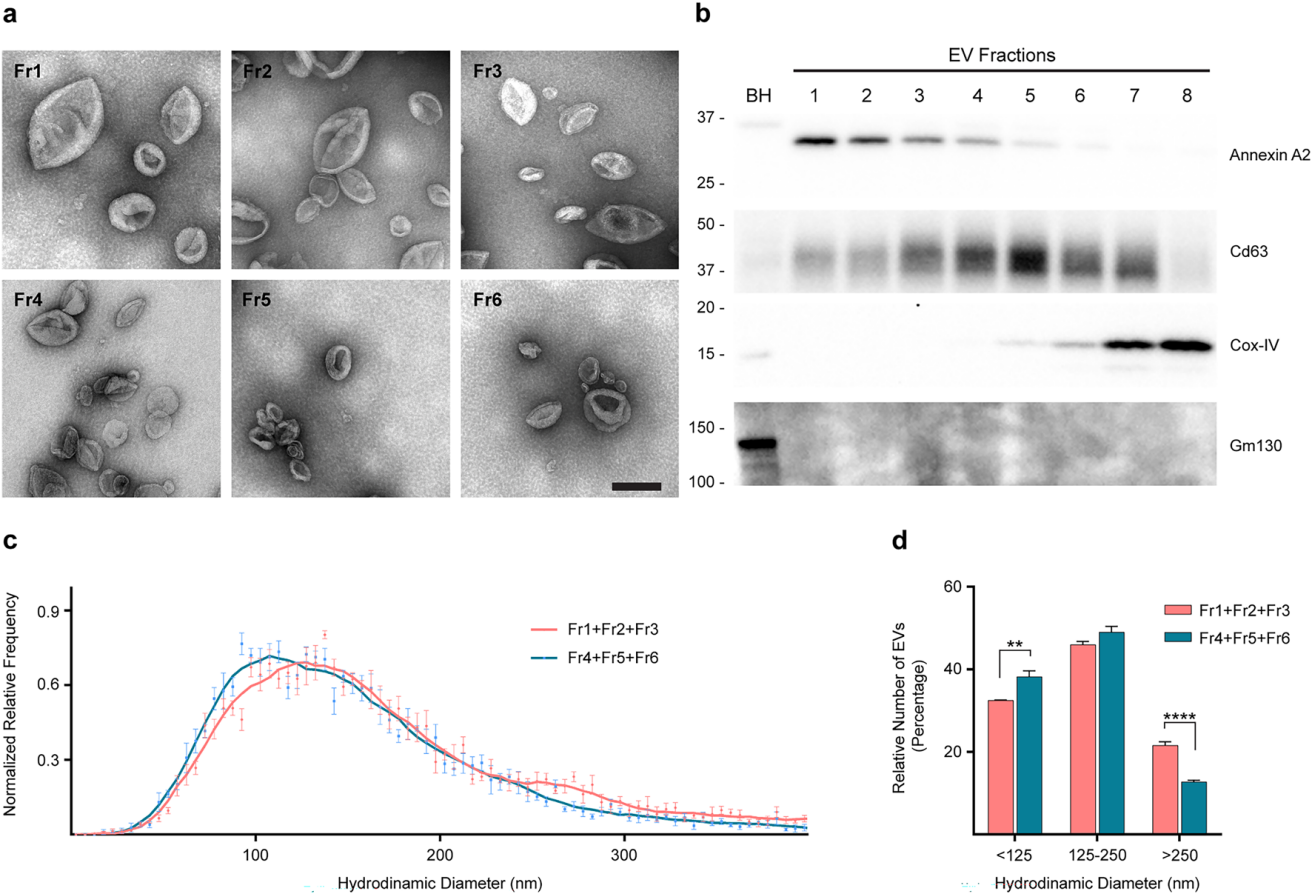
Characterization of EVs isolated from mouse brains. **a** Representative photomicrographs imaged by transmission electron microscopy of brain EV fractions 1 to 6, fractionated by an OptiPrep density gradient. Scale bar: 200 nm. **b** Representative Western blot analyses of EVs isolated from the brain of a 3-month-old wild-type mouse and fractionated by an OptiPrep density gradient. The same volume of each fraction was loaded in each lane. Annexin A2, a marker of microvesicles is enriched mainly in fractions 1 to 3, the exosomal marker Cd63 in fractions 4 to 6, the marker of EVs of mitochondrial origin (mitovesicles) Cox-IV is found mainly in fractions 7 and 8, and a marker of an unrelated intracellular compartment (Gm130) was not observed in any of the EV fractions but found in brain homogenates (BH). **c** Diameter analysis of brain EVs by NTA. The frequency of the distribution was normalized to the mode, while the bell curves were obtained using a four-point moving average. The numbers of fractions 1 to 3 and of fractions 4 to 6 were combined as they showed similar characteristics (*n* = 3 mice per group). **d** Percentage of vesicles with a diameter that falls within a 125 nm bin, as estimated in **c** (*n* = 3 mice per group). Statistical test: two-way ANOVA with Bonferroni’s multiple comparisons test. Data are represented as mean ± SEM. ** *P* < 0.01, **** *P* < 0.0001

EVs were isolated from the right hemibrain of mice of the eight groups: females, males, ovariectomized and sham operated females, treated with either cocaine or saline. EV subpopulations were fractionated using an iodixanol-based density-gradient which enriches microvesicles in fractions 1, 2, and 3, and exosomes in fractions 4, 5, and 6 [24]. The morphology of the EV species in each of the six fractions studied here was identified by transmission electron microscopy, which revealed that all fractions contained characteristic cup shaped EVs (Fig. 4a). None of the samples contained debris, broken membranes, or protein aggregates, an indication of their purity. Western blot analyses were performed to identify the type of vesicles contained in each EV fraction. An antibody to Annexin A2, a marker of microvesicles, identified these plasma membrane-derived vesicles mainly in fractions 1–3 (Fig. 4b). The tetraspanin Cd63, a general marker of exosomes, was enriched in fractions 4–6, consistent with our previous reports. Fraction 8 contained mainly EVs of mitochondrial origin (mitovesicles), not considered in this study, while fraction 7 was promiscuous, containing both exosomes and mitovesicles (Fig. 4b). Proteins of unrelated intracellular compartments, such as the Golgi marker Gm130, were not observed by Western blot analyses in any of the fractions, confirming a lack of intracellular contaminants (Fig. 4b). Nanoparticle tracking analysis (NTA) demonstrated that EVs found in fractions 1–3 were larger than EVs found in fractions 4–6 (Fig. 4c, d), consistent with enrichment of microvesicles and exosomes, respectively.

**Fig. 5:**
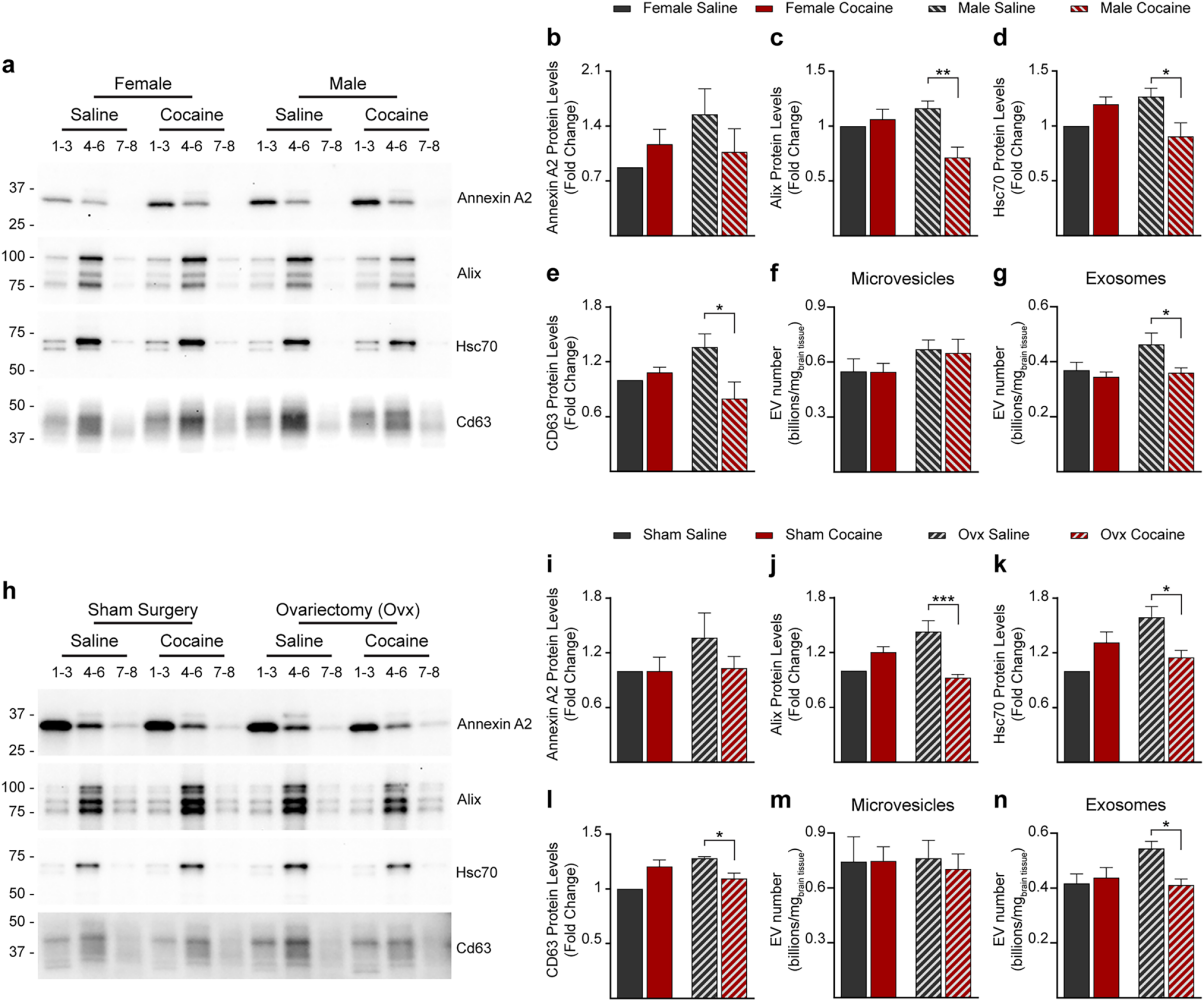
Cocaine does not affect the level of EVs in the brain of female mice but reduces the levels of EVs in the brain of male mice. **a** Representative western blot analyses of female and male EV lysates upon treatment with either cocaine or saline. Markers of microvesicles and exosomes were assessed. The same volume of the EV fractions was loaded in each lane. Scanned bands were quantified and normalized to hemibrain weight for Annexin A2 (**b**), Alix (**c**), Hsc70 (**d**) and Cd63 (**e**) (*n* = 5 mice per group). **f** and **g** Number of EVs found in microvesicle-enriched fractions (fractions 1 + 2 + 3) (**f**) and in exosome-enriched fractions (4 + 5 + 6) (**g**) of EVs isolated from the brain of female and male mice upon treatment with either cocaine or saline, as quantified by NTA and normalized to hemibrain weight (*n* = 5 mice per group). **h** Representative western blot analyses of EV lysates from females following sham surgery and ovariectomized females upon treatment with either cocaine or saline. Markers of microvesicles and exosomes were assessed. The same volume of the EV fractions was loaded in each lane. Scanned bands were quantified and normalized to hemibrain weight for Annexin A2 (**i**), Alix (**j**), Hsc70 (**k**), and Cd63 (**l**) (*n* = 5 mice per group). **m** and **n** Number of EVs found in microvesicle-enriched fractions (fractions 1 + 2 + 3) (**m**) and in exosome-enriched fractions (4 + 5 + 6) (**n**) of EVs isolated from the brain of female mice following sham surgery and ovariectomized females upon treatment with either cocaine or saline, as quantified by NTA and normalized to hemibrain weight (*n* = 5 mice per group). Fractions 1–3 are enriched in microvesicles, fractions 4–6 are enriched in exosomes, fractions 7–8 are enriched in other EVs. Statistical test: two-way ANOVA with Bonferroni’s multiple comparisons test. Data are represented as mean ± SEM. * P < 0.05, ** P < 0.01, and *** P < 0.001

Western blot analyses of the EV fractions isolated from female brains with the microvesicle marker Annexin A2 and with the exosomal markers Alix, Tsg101, Hsc70, and Cd63 did not identify any effect of cocaine on the levels of exosomes and microvesicles. However, the level detected by Western blot analyses for exosomes (but not microvesicle) was reduced by cocaine in males (Fig. 5a–e). These data suggest a male-specific reduction in exosome generation upon cocaine administration and are consistent with the respective endosomal changes. To validate this speculation, we performed NTA to quantify the number of EVs. NTA showed that while cocaine does not affect the number of EVs in the brains of female mice, it causes a significant reduction in the number of exosomes in the brains of males (Fig. 5f, g). Cocaine also reduced the number of exosomes in the brain of ovariectomized mice, mimicking its effect in males, as estimated by western blot and NTA analyses (Fig. 5h–n).

**Fig. 6:**
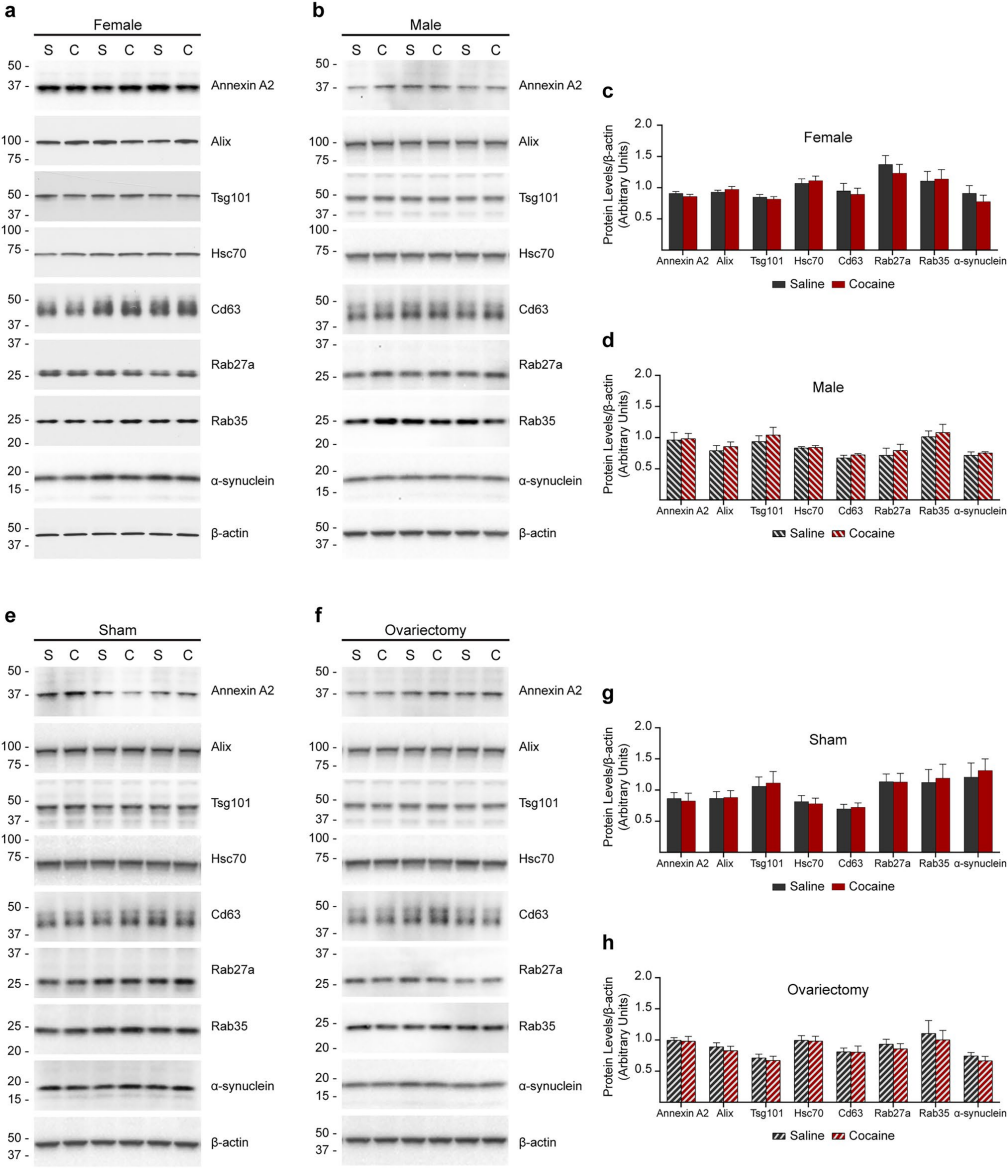
Cocaine injection does not affect the level of expression of EV protein markers and of α-synuclein. **a**–**h** Western blot analyses of brain homogenates of female (**a**), male (**b**), and females undergone sham (**e**) or ovariectomy (**f**) surgeries injected with either saline (S) or cocaine (C) (*n* = 7 mice for the female set, 6 for the other groups). The antibodies detected a marker of microvesicles (Annexin A2), markers of exosomes (Alix, Tsg101, Hsc70, Cd63, Rab27a, and Rab35), and α-synuclein. β-actin was used as a loading control. The same amount of total protein was loaded in each lane. Graphs in **c**, **d**, **g** and **h** show the densitometric quantification of the bands normalized to β-actin in the same lane. Statistical test: two-way ANOVA with Bonferroni’s multiple comparisons test. No difference between cocaine and saline treatments was found. Data are represented as mean ± SEM

Western blot analyses of homogenates of the left hemibrains of the same mice revealed that cocaine injection did not affect the level of expression of any of these proteins in either females, males, sham-operated, or ovariectomized mice (Fig. 6). In addition, normalization of the amount of each of the proteins investigated to the total protein amount within each fraction did not reveal an effect of cocaine on the protein levels (data not shown), indicating that it is not the level of expression or loading of EVs that are affected by cocaine in males and in ovariectomized females. These data show that the reduction in the number of exosomes identified by protein markers is due to either reduced production, reduced secretion, or increased uptake and turnover of exosomes in the brain of males.

### Cocaine Impacts the Level of α-Synuclein in Brain-EVs Differently in Males and Females

**Fig. 7:**
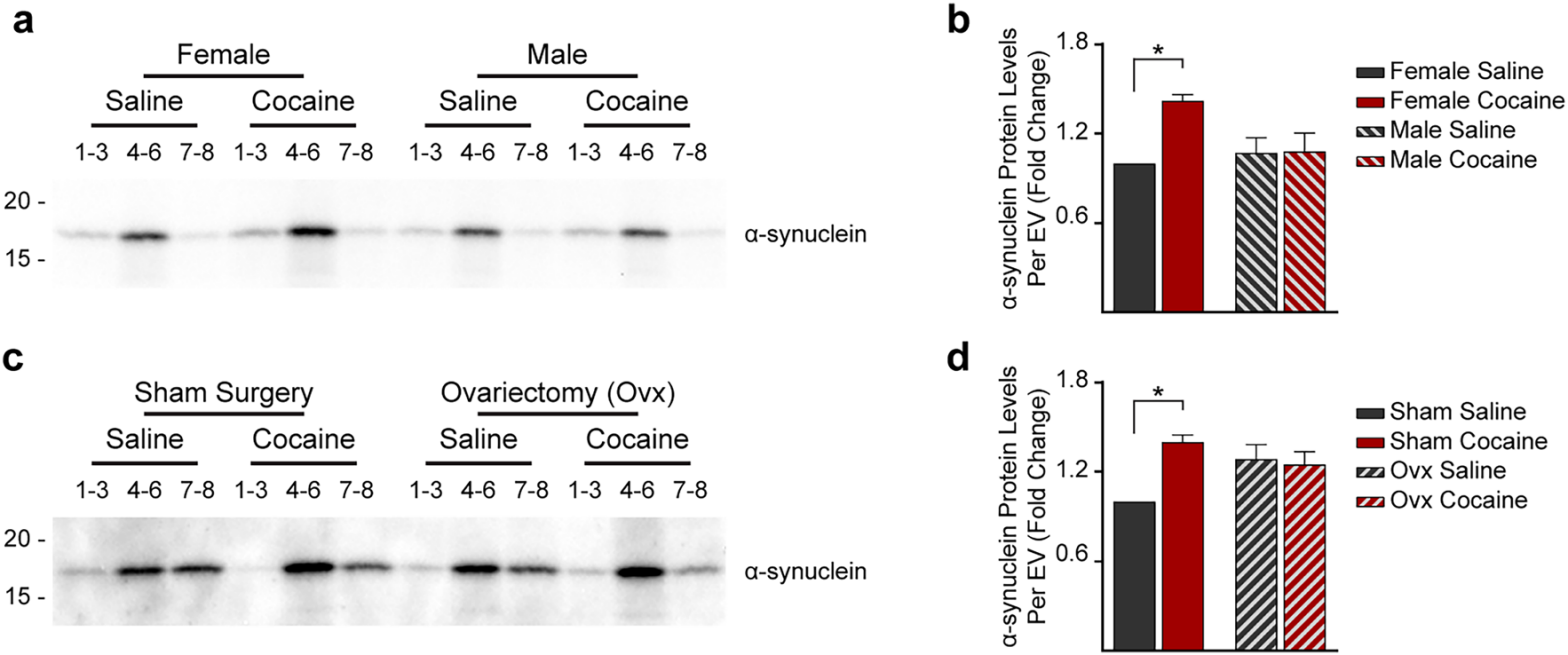
Cocaine increases the content of α-synuclein in exosomes in the brain of females. **a**–**d** Western blot analysis of α-synuclein content in EV fractions (*n* = 5 mice per group). The same volume of the EV fractions was loaded in each lane. Scanned bands were quantified and normalized to the total EV protein content as estimated by the BCA assay for females and males (**a**-**b**), females following sham surgery, and ovariectomized females (**c**-**d**). Fractions 1–3 are enriched in microvesicles, fractions 4–6 are enriched in exosomes, fractions 7–8 are enriched in other EVs. Statistical test: two-way ANOVA with Bonferroni’s multiple comparisons test. Data are represented as mean ± SEM. * P < 0.05

Given that cocaine- and DA-induced behaviors related to chronic substance exposure as well as MVB homeostasis are mediated by α-synuclein [3–6] we investigated the effect of cocaine treatment on the level of expression and EV content of α-synuclein in the brain of male and female mice. α-synuclein amount per vesicle was quantified by Western blot analysis of the different exosomal fractions and normalized to the protein content in each fraction. The data showed that the α-synuclein content is higher in exosomes in the brain of cocaine-injected as compared with saline-injected females. No effect of cocaine on the level of α-synuclein per exosome was observed in the brains of males (Fig. 7a, b). Ovariectomy eliminated the increase in the amount of α-synuclein in brain exosomes of females treated with cocaine as compared with saline-injected females, mimicking the male response (Fig. 7c, d). Western blot analysis of homogenates of the left hemibrains of the mice revealed that cocaine injection did not affect the level of overall brain α-synuclein expression (Fig. 6). Thus, cocaine affects the loading of exosomes with α-synuclein differently in males and females. While cocaine reduces the number of exosomes in the brain of males, it increases the content of α-synuclein per exosome, without affecting the number of total exosomes, in the brain of females.

## Discussion

### Sex Differences in Cocaine Abuse Disorders

Cocaine abuse disorders are observed in both men and women; however, compared to men, women have a higher likelihood of becoming addicted at a younger age [35], take higher doses [36], require less time to become addicted [37], experience more cravings [38, 39], and have a greater difficulty remaining abstinent [40–42]. Sex differences in response to cocaine are also seen in experimental animals [39]. It was proposed that the differential efficacy of cocaine across sexes is driven by circulating estrogens, which affect DAT activity [43]. In females, behavioral and physiological responses to cocaine have been correlated to menstrual cycle fluctuations of estrogens. During the follicular phase (rising estrogens levels), females experience increased potency of cocaine, compared to luteal phase (low estrogens levels) [43–45], and females subjectively reported heightened cravings [46, 47]. Additionally, prepubescent rats did not show sex differences in cocaine-induced locomotor activity after one or repeated cocaine injections [48], suggesting that differences in gonadal hormones of adult animals mediate, at least partially, the sex difference in response to cocaine.

Consistent with these previous studies, we observed a greater locomotor activity response in female C57BL/6 mice than males and that, similar to males, ovariectomized females had less locomotor activity response to cocaine than sham treated female mice. In addition to behavioral differences, we found sex differences within the endosomal and exosomal pathways and ovariectomy undid this difference.

### The Effect of Cocaine on the Endosomal-Exosomal Pathways

It was previously shown that cocaine causes alterations in the endosomal, autophagic, and lysosomal system, both in vitro and in vivo in selected populations of neurons, although sex differences have not been examined [12–14]. We find that the number and area per neuron of early and late endosomes are higher in brains of male as compared to female mice at three months of age, a previously unappreciated sex difference in the neuronal endosomal pathway. Cocaine administration for twelve days at that age did not affect the endosomal pathway in cortical neurons of female mice. However, consistently with sex-differences in cocaine effects, this cocaine treatment leads to a decrease in the number and area per neuron of early and late endosomes in cortical neurons of male mice. This sex-dependent effect was profound, with the neuronal early and late endosome area and number reaching lower levels in treated males compared to females, regardless of treatment status. Additionally, ovariectomized females showed the male pattern for neuronal endosomes: greater endosome number and area fraction per neuron at baseline, with a decrease in the number of early and late endosomes following cocaine injection, arguing that the sex difference is mediated by female sex hormones.

While cocaine affects the secretion of EVs studied in vitro, the reported effect depended upon the cell type studied, the concentration of cocaine used, and the experimental conditions. Although cocaine treatment of human glioblastoma cell cultures increased the number of EVs, mainly exosomes, in the medium in a concentration-dependent manner [49], there was a significant decrease in the number of total EVs secreted by BV2 microglial cells after cocaine treatment [50]. In addition to effects on EV and exosome secretion, it was shown that self-administration of cocaine reduces the internalization of neuronal exosomes, particularly by astrocytes in the nucleus accumbens, but not in the motor cortex [51]. In order to capture a comprehensive picture using an in vivo model in which sex-differences can also be explored, we examined microvesicles and exosomes in the brain extracellular space. Similar to our endosomal findings, cocaine did not affect the level of EVs in the brain extracellular space of female mice. However, cocaine treatment reduced the level of exosomes in the brain of male mice. Again, ovariectomized females resembled males in the brain exosome response to cocaine injection.

Thus, our data suggest that hormonal regulation of the endosomal-exosomal pathways may be one mechanism responsible for the sexually dimorphic responses to cocaine. Given the sex-effects on chronic cocaine exposure described above, it is striking that brain endosomal and exosome responses to cocaine are similarly sex-dependent. While this may be correlative, many lines of research would suggest that changes within the endosomal system might contribute to effects induced by chronic cocaine exposure [7–9, 52–54].

Interdependence between the endosomal and exosomal systems were previously shown to occur in an age-dependent manner. A dysfunctional endosomal pathway and abnormally enlarged early and late endosomes in neurons are an early characteristic of Down syndrome and in the trisomy mouse model Ts[Rb(12.17^16^)]2Cje [21, 55]. An age-dependent increase in exosome levels was found in the brain extracellular space of 12-month-old Ts2 mice as compared to diploid littermates, but not in younger, 3- and 8-month-old mice, suggesting a compensatory role for exosomes in the regulation of endosomal function in Down syndrome [20]. In human post-mortem tissue and mouse models humanized for apolipoprotein E, apolipoprotein E4, the greatest genetic risk factor for Alzheimer’s disease, drives an age-dependent lowering of the exosome levels in the brain extracellular space. While not present at 6 months of age, it is detectable at 12 months in apolipoprotein E4 murine carriers. This reduction in brain exosome levels occurs earlier than endosomal changes that initiate at 18 months of age, arguing that an apolipoprotein E4-driven failure in exosome production plays a primary role in endosomal and lysosomal deficits that occur in apolipoprotein E4 mouse and human brains [15]. Here we report for the first time a change in both the endosomal and exosomal systems upon cocaine administration, occurring together in a short period of time in young mice.

### The Role of α-Synuclein Transfer in Extracellular Vesicles

Multiple studies have shown the association of α-synuclein with EVs and the transfer of the protein between cells via EVs [56, 57]. It was also demonstrated that neuroblastoma cell-derived EVs containing α-synuclein induce cell death in neuronal cells [58]. Inhibition of lysosomal function in α-synuclein overexpressing neural cell lines increased exosomal secretion of α-synuclein and promoted cell-to-cell transfer of the protein [56]. Furthermore, blockage of macroautophagy increased exosomal secretion of α-synuclein [59].

We found that more α-synuclein is incorporated into exosomes in female mice upon cocaine administration, while no effect was found in the EVs isolated from treated male mice. These findings reveal that recruitment of α-synuclein is sex specific and incorporation of α-synuclein may provide a novel mechanism for the heightened cocaine cravings and sensitivity of females as compared with males.

## Conclusion

**Fig. 8:**
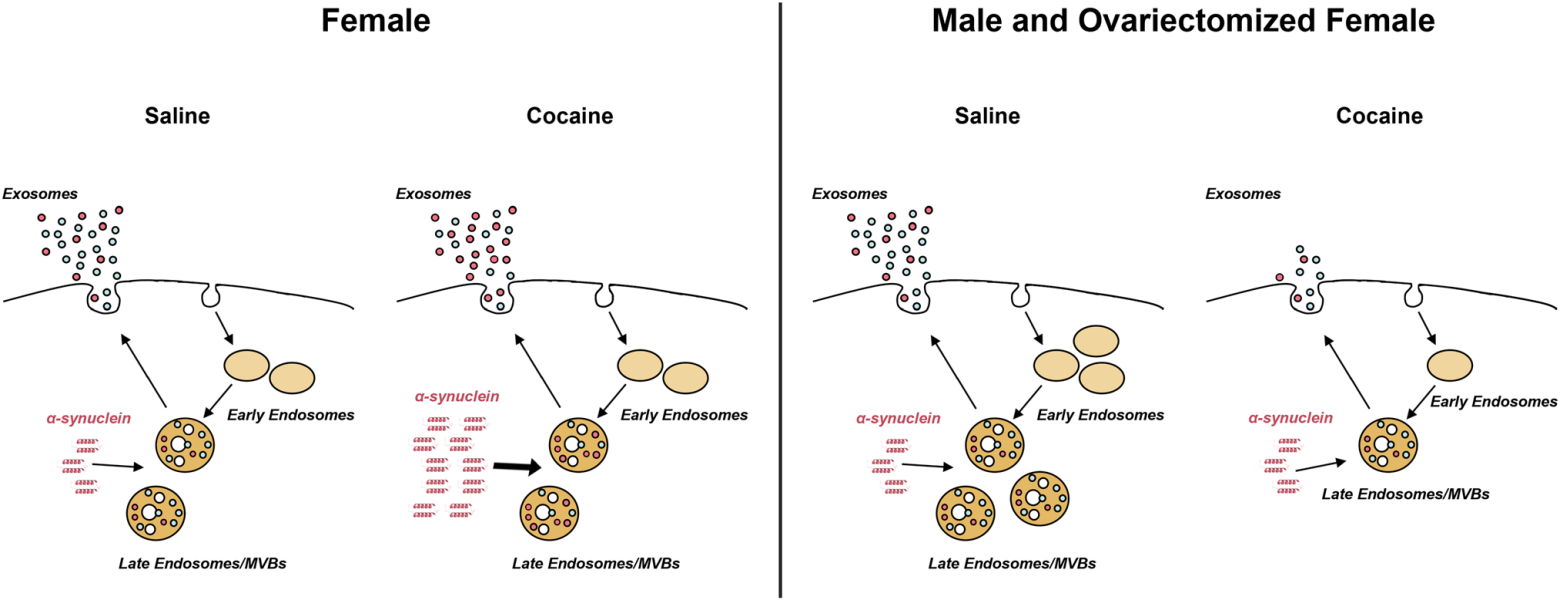
Schematic representation of the endosomal and exosomal pathways in cocaine treated female and male mice. Endocytosed material in the cell is transported to early endosomes, that eventually mature to become MVBs. The invagination of the MVBs membrane results in the formation of intraluminal vesicles, which are released into the extracellular space upon fusion of MVBs with the plasma membrane. Additional material is released from the cell by formation of microvesicles from the plasma membrane and mitovesicles from mitochondria (not shown in this figure). Cocaine reduces the number of neuronal early and late endosomes, as well as exosomes in the brain of male but not female mice. Cocaine also enhances the loading of α-synuclein into exosomes in the brain of female but not male mice, resulting in a higher spreading of α-synuclein to neighboring cells. Red-filled circles: intraluminal vesicles/exosomes loaded with α-synuclein. Cyan-filled circles: intraluminal vesicles/exosomes negative for α-synuclein

In sum, we found that the number of EVs released into the brain extracellular space of male mice is reduced following cocaine administration (Fig. 8). While the number of EVs released in the brain of female mice is not affected by cocaine, the secreted exosomes contain larger amount of α-synuclein (Fig. 8). We hypothesize that higher content of α-synuclein in exosomes causes higher level of uptake of the protein by naïve cells, potentially propagating cocaine-induced abnormalities throughout the brain. Our findings that females secrete larger amounts of α-synuclein through exosomes than males suggest a mechanism for the higher susceptibility of females to cocaine abuse.

## Data Availability

All data generated or analyzed during this study are included in this published article. This study did not generate new unique reagents. No custom code was used in this study. No large datasets were generated. The published article includes all the analyses generated during this study.
